# Gambling by Young Adults in the UK During COVID-19 Lockdown

**DOI:** 10.1007/s10899-021-10029-y

**Published:** 2021-05-17

**Authors:** Alan Emond, Agnes Nairn, Sharon Collard, Linda Hollén

**Affiliations:** 1 Centre for Academic Child Health, Bristol Medical School, University of Bristol, 1-5 Whiteladies Road, Bristol, BS8 1NU UK; 2 School of Management, Faculty of Social Science and Law, University of Bristol, Bristol, UK; 3 School of Geographical Sciences, University of Bristol, Bristol, UK

**Keywords:** Gambling, Lockdown, COVID-19, ALSPAC

## Abstract

Gambling is a common activity amongst young adults in the UK, and was a behavior of interest during the early mitigation against COVID-19 (first lockdown). The Avon Longitudinal Study of Parents and Children (ALSPAC) was used to investigate attitudes, moods and behavior during lockdown in England. ALSPAC participants were invited to complete online questionnaires in May 2020, including a set of questions about frequency of gambling and gambling activities which had been asked three years previously. Mental health and wellbeing data and alcohol use were also collected as part of lockdown questionnaires. Gambling questions were completed by 2632 young adults, 71% female, with a mean age of 27.8 years. Overall, gambling frequency reduced during lockdown for both males and females, but more males engaged in regular (weekly) gambling. Gambling activities became more restricted compared to previous reports, but online gambling (e.g. online poker, bingo, casino games) was more frequent. Previous gambling behaviour predicted gambling frequency during lockdown. No associations were apparent between gambling frequency and measures of mental health and well-being. Heavy alcohol use was strongly linked with regular gambling during lockdown. Gamblers were more than twice as likely as non-gamblers to have experienced financial difficulties pre-COVID, but gambling frequency was not related to employment status during lockdown. Online gambling increased during lockdown, whilst offline gambling activities decreased in frequency. A small minority of regular weekly gamblers, who tended to be male and heavy users of alcohol, participated in a wide range of online and offline gambling activities.

## Introduction

In the UK, gambling is a common activity amongst young adults. In the Health Survey for England in 2018 (NHS Digital, 2019), 57% of adult men and 54% of adult women reported gambling in the past year. In the age range 16–24 years, 45% of men and 33% of women reported gambling in the past year, and 20% of men and 2% of women gambled online. The increased availability of gambling and the expansion of opportunities to gamble online have led to increases in the number of young people who gamble on a regular basis (Bray et al 2014; Delfabbro et al., 2014; Killick & Griffiths, 2019; Gambling Commission, 2019).

Mitigation strategies against COVID-19 resulted in a comprehensive set of restrictions on normal life in the UK (termed ‘lockdown’) from 23rd March until the beginning of July 2020. During this period, many people were confined to their homes, with impacts on their physical and mental health and personal relationships. To capture the impact of lockdown, it was important for longitudinal studies to mount ‘real- time’ data collection and given its prevalence in the UK population (NHS Digital, 2019), gambling was one important behavior to investigate. During lockdown physical gambling premises were required to shut, but national lottery tickets and scratchcards could still be purchased from stores (as well as online), and online gambling continued as before, although in-game betting was limited by the lack of live sport.

The Avon Longitudinal Study of Parents and Children (ALSPAC), a three generation cohort based in the SW of England (http://www.bristol.ac.uk/alspac), was able to collect repeat data quickly during lockdown using an existing infrastructure for online data collection (Northstone et al., 2020). The young people in ALSPAC had previously been asked about their gambling activities at the age of 24 years as part of a longitudinal investigation of gambling in the transition from adolescence to young adulthood (Hollén et al., 2020). Aged 24 years, 66% reported gambling in the last year, with a heavy male bias in the 17% of participants admitting regular weekly gambling. The same gambling questions were asked of the young adults (mean age 27.8 years) in the online questionnaire during lockdown. Previous research suggests that young adult men are a group particularly likely to use the internet for gambling (Calado et al 2017; Emond et al., 2020), and those young men that gamble regularly have a higher rate of mental health problems (Petry & Weinstock, 2007) and of abusing alcohol (French et al., 2008)—so this group were of special interest for this gambling research carried out in lockdown. The hypothesis to be tested was that during lockdown young males in particular would engage in more frequent gambling online compared to their previously reported gambling behaviours.

## Methods

ALSPAC is an intergenerational longitudinal cohort study that recruited pregnant women residing in Avon, UK with expected dates of delivery 1st April 1991 to 31st December 1992. The initial cohort consisted of 14,541 pregnancies resulting in 14,062 live births and 13,988 children who were alive at 1 year of age. From the age of seven onwards, the initial sample was bolstered with eligible cases who had originally failed to join the study and there were subsequently 14,701 children alive at 1 year of age following this further recruitment (Boyd et al., 2013). An update on the index children in ALSPAC has recently been published (Northstone et al., 2019). The study website contains details of all the data that are available through a fully searchable data dictionary and variable search tool (http://www.bristol.ac.uk/alspac/researchers/our-data/).

ALSPAC participants were sent two extra online questionnaires during lockdown- the first in April 2020 and the second at the end of May 2020. The questionnaires were developed and deployed using REDCap (Research Electronic Data CAPture tools (Harris et al., 2009); a secure web application for building and managing online data collection exercises, hosted at the University of Bristol.

The gambling questions were included in the second questionnaire, the details of which have been published as a Data note (Northstone et al., 2020). The second online questionnaire, asking about physical and mental health, lifestyle and behaviours, employment and finance, was deployed across the parent and offspring generations between the 26th May and 5th July 2020. The response rate was 44%, resulting in 2711 young adults completing the questionnaire.

The gambling questions were similar to those previously asked to the same cohort when aged 24 years (Hollén et al., 2020), which were originally derived from the British Gambling Prevalence Survey (Wardle et al., 2008). However, whereas previous gambling questionnaires included 17 questions on what types of gambling activities participants took part in, the COVID questionnaire was reduced to 8 questions about participation in last month with regards to: national lottery, scratch cards, online gambling, online betting, betting exchange, spread betting, private betting, other form of gambling (Appendix). Response options were: not at all, less than weekly, every week or every day/almost every day. Occasional gamblers were defined as those who gambled less than weekly, and regular gamblers as those who gambled weekly or more frequently.

Questions on mental health and wellbeing included specific questions about worries during the pandemic. Depression was assessed using the Short Moods and Feelings Questionnaire (SMFQ) (Ancold & Stephen, 1995) and anxiety using the General Anxiety Disorder-7 questionnaire (GAD-7) (Spitzer et al., 2006). Participants’ wellbeing was self-reported using the Warwick-Edinburgh Mental Wellbeing Scales (WEMWBS) (Tennant et al., 2007). Alcohol use was self-reported with the AUDIT Tool (Bush et al., 1998).

Employment status during lockdown was reported in the following categories: “working/studying same or more hours”, “working/studying reduced hours/on paid leave/on furlough”, “not in paid work/unpaid leave”, “self-employed currently working”, “self-employed not currently working”. Participants were asked about how they managed financially pre-COVID and reported as “living comfortably”, “doing all right”, “just about getting by” and “finding it quite/very difficult”.

### Consent

Completion of the lockdown questionnaires was optional and choosing to complete the questionnaire is considered informed consent for the questionnaire. Ethical approval for the study was obtained from the ALSPAC Ethics and Law Committee and the Local Research Ethics Committees. Informed consent for the use of data collected via questionnaires and clinics was obtained from participants following the recommendations of the ALSPAC Ethics and Law Committee at the time. Study participants have the right to withdraw their consent for elements of the study or from the study entirely at any time. Full details of the ALSPAC consent procedures are available on the study website (http://www.bristol.ac.uk/alspac/researchers/research-ethics/).

### Statistical Analyses

All analyses were conducted using Stata v.15.1. Chi-square tests, two-proportion z-tests, ANOVAs, binary logistic regressions or multinomial logistic regressions were used.

## Results

### Sample

The second ALSPAC COVID-19 questionnaire was complete by 2711 young adults, with a mean age of 27.8 years (SD: 0.6). The population who responded were predominantly white (> 96%) and 80% had at least A-level qualifications. Of these respondents, 2632 (97.1%) answered the gambling questions: 771 males (29.3%) and 1861 (70.7%) females.

Previously 4304 ALSPAC participants, with a mean age of 24.9 years (SD: 0.6), had answered the 24-year gambling questionnaire: 1501 males (34.9%) and 2803 (65.1%) females. The female preponderance was more marked in the respondents to the COVID-19 questionnaire than in the 24 year sample (p < 0.001). The bias introduced by this female preponderance in the COVID-19 questionnaire probably resulted in an underestimate of the number of regular gamblers.

Of those individuals that answered the gambling questionnaire at 24 years, (N = 4304), 3872 (90%) were sent the COVID questionnaire but only 2160 (56%) answered the questionnaire. Those that did not answer the COVID questionnaire were more likely to be male, have a lower IQ (measured at age 8), have slightly raised hyperactivity scores (measured at age 16.5), were more likely to have a diagnosis of moderate/severe alcohol use disorder (measured at age 22) and more likely to have smoked 100+ cigarettes in their life time (measured at age 25). There was no influence on whether the questionnaire was answered or not of regular gambling at age 24, employment status at age 20, or if there was a history of maternal gambling.

### Gambling Frequency

The frequency of gambling during the COVID-19 lockdown, compared with the frequency previously reported at 24 years, is presented in Table 1. Overall, gambling frequency reduced during lockdown for both males and females (< weekly and >  = weekly: p < 0.001 both sexes). The gender difference widened in those who engaged in regular gambling, with males nearly 3 times more likely than females to gamble regularly.

**Table 1:** Gambling Frequency by ALSPAC participants during COVID lockdown and at age 24 years

	COVID-19 questionnaire		24-year questionnaire	
	Males (n = 771)	Females (n = 1861)	Males (n = 1501)	Females (n = 2803)
No gambling	614 (79.6%)	1693 (91.0%)	490 (32.6%)	1202 (42.9%)
< weekly gambling	91 (11.8%)	115 (6.2%)	773 (51.5%)	1391 (49.6%)
> = weekly gambling	66 (8.6%)	53 (2.9%)	238 (15.9%)	210 (7.5%)

Gambling at 24 years strongly predicted whether people gambled or not during lockdown irrespective of gender (OR (95% CI): 10.70 (6.57, 17.44), n = 2112). Using the sub-sample of individuals who gambled at both times (n = 1255) showed that a larger proportion of males compared to females did not change their frequency of gambling (p < 0.001) whereas a larger proportion of females decreased their gambling frequency during lockdown (p < 0.001). The proportion that increased their gambling frequency did not differ between males and females (p = 0.28; Table 2).

**Table 2:** Correlation of gambling frequency at 24 years and during COVID lockdown

Frequency during COVID	Males	Females	Total
Decreased frequency	303 (77.9%)	746 (86.1%)	1049 (83.6%)
Stable frequency	64 (16.5%)	83 (9.6%)	147 (11.7%)
Increased frequency	22 (5.7%)	37 (4.3%)	59 (4.7%)
Totals	389 (100%)	866 (100%)	1255

### Gambling Activities

The different gambling activities reported in the 24 year and COVID questionnaires are presented in Table 3, which shows a different pattern for regular (weekly or more frequent) gamblers compared to occasional (less than weekly) gamblers. For regular gamblers, a few activities, including national lottery, private betting and online gambling, increased during COVID-19 lockdown compared to what was reported 3 years previously. The proportion gambling on other activities remained stable. In contrast, for occasional gamblers, most gambling activities decreased in frequency or remained stable, with the exception of online gambling which increased in frequency for both occasional and regular gamblers. Online gambling included online poker, bingo, casino games. Online betting, involving placing bets on any event or sport, including e-sports, overall remained at similar rates to 24 years (although most UK based sporting events were cancelled during the lockdown).

**Table 3:** Gambling Activities reported in the COVID-19 and 24-year questionnaires

	Gambling frequency		
	Not at all	< weekly	> = weekly
**National lottery**			
COVID (n = 320)	145 (45.3%)	106 (33.1%)	69 (21.6%)
Age 24 (n = 2607)	939 (36.0%)	1487 (57.0%)	181 (6.9%)
P-value	0.001	< 0.001	< 0.001
**Scratch cards**			
COVID (n = 316)	206 (65.2%)	90 (28.5%)	20 (6.3%)
Age 24 (n = 2605)	904 (34.7%)	1568 (60.2%)	133 (5.1%)
P-value	< 0.001	< 0.001	0.36
**Online gambling**			
COVID (n = 320)	215 (67.2%)	74 (23.1%)	30 (9.7%)
Age 24 (n = 2579)	2235 (86.7%)	310 (12.0%)	34 (1.3%)
P-value	< 0.001	< 0.001	< 0.001
**Online betting**			
COVID (n = 321)	221 (68.9%)	78 (24.3%)	22 (6.8%)
Age 24 (n = 2594)	1798 (69.3%)	651 (25.1%)	145 (5.6%)
P-value	0.87	0.76	0.36
**Betting exchange**			
COVID (n = 319)	313 (98.1%)	< 5 (1.3%)	< 5 (0.6%)
Age 24 (n = 2585)	2476 (95.8%)	96 (3.7%)	13 (0.5%)
P-value	0.04	0.02	< 0.001
**Spread betting**			
COVID (n = 319)	314 (98.4%)	< 5 (0.9%)	< 5 (0.6%)
Age 24 (n = 2579)	2533 (98.2%)	42 (1.6%)	< 5 (0.2%)
P-value	0.78	0.35	0.08
**Private betting**			
COVID (n = 322)	279 (86.6%)	35 (10.9%)	8 (2.5%)
Age 24 (n = 2586)	2083 (80.5%)	490 (19.0%)	13 (0.5%)
P-value	0.008	< 0.001	< 0.001
**Other gambling**			
COVID (n = 322)	303 (94.1%)	10 (3.1%)	9 (2.8%)
Age 24 (n = 2559)	2489 (97.3%)	60 (2.3%)	10 (0.4%)
P-value	0.002	0.40	< 0.001

Sample size represents those that said they have gambled on one or more activities

### Gambling and Mental Health

Anxiety levels reported by the cohort during lockdown were generally high (Table 4), but there did not appear to be a relationship between anxiety levels and gambling frequency (χ^2^ = 5.8, p = 0.45).

**Table 4:** Anxiety levels, measured using the GAD-7, and gambling frequency [N (row%)]

	No anxiety (score 0–4)	Mild anxiety (score 5–9)	Moderate anxiety (score 10–14)	Severe anxiety (score 14–21)	Totals
Did not gamble	861 (46.4%)	551 (29.7%)	278 (15%)	165 (8.9%)	1855 (100%)
Occasional gamblers	83 (50.9%)	35 (21.5%)	29 (17.8%)	16 (9.8%)	163 (100%)
Regular gamblers	47 (51.1%)	25 (27.2%)	13 (14%)	7 (7.6%)	92 (100%)
Totals	991 (47%)	611 (29%)	320 (15.2%)	188 (8.9%)	2110 (100%)

Around 15% of participants reported depressive symptoms during lockdown (Table 5), but likelihood of depression was not associated with gambling frequency (χ^2^ = 1.4, p = 0.50).

**Table 5:** Probability of depression, measured on the SMFQ, and gambling frequency

	Unlikely depression (score < 12)	Likely depression (score 12+)	Totals
Did not gamble	1553 (84.6%)	283 (15.4%)	1836 (100%)
Occasional gamblers	133 (82%)	29 (17.9%)	162 (100%)
Regular gamblers	78 (87.6%)	11 (12.4%)	89 (100%)
Totals	1764 (84.5%)	323 (15.5%)	2087 (100%)

Wellbeing scores (mean [SD]) during lockdown were similar in non-gamblers (44 [8.5]), occasional gamblers (43 [8.8]), and regular gamblers (44 [8.7]; p = 0.20).

### Gambling and Alcohol Use

Although frequency of drinking any alcohol (< weekly or >  = weekly) during lockdown did not show an association with gambling frequency (p = 0.38), frequency of heavy drinking did (p < 0.001). Participants drinking more than 6 units on one occasion regularly (weekly or more) were more likely to be male [OR (95% CI): 1.62 (1.31, 2.01)] and 2.38 times (CI: 1.56, 3.63) more likely to gamble weekly during lockdown compared to not gambling at all. The odds ratio for less than weekly gambling was 1.83 (1.30, 2.56), no different to that for weekly gambling (p = 0.31). The association between heavy alcohol intake and less than weekly gambling was only significant in males [2.67 (1.66, 4.30); females: 1.04 (0.61, 1.78)], whereas the association with weekly or more frequent gambling was evident in both sexes [males: 2.03 (1.15, 3.58); females: 2.42 (1.28, 4.61)].

### Gambling and Personal Financial Situation

Those that struggled financially before COVID were more likely to answer yes to *any* gambling during lockdown (< weekly gambling and >  = weekly gambling combined due to small numbers). Odds ratio for those reporting “just getting by” pre-COVID and any gambling was 1.56 (95% CI: 1.07, 2.26) and 2.23 (1.23, 4.06) for those reporting “finding it quite or very difficult”.

No relationship was apparent between gambling frequency and current employment status during lockdown (p = 0.63).

## Discussion

This study of a well-characterised group of young adults during COVID-19 mitigation has captured in ‘real time’ their mood and gambling behavior. As expected, the overall frequency of gambling reduced during lockdown because of the restrictions on movement, but the activity which could be done from home- online gambling- did increase in both sexes. The main hypothesis, that during lockdown young males in particular would engage in more frequent gambling, was supported. Regular gamblers not only gambled online more than previously, but also betted on games at home and gambled on lotteries and using scratchcards more than reported at 24 years. Regular weekly gambling was associated with heavy use of alcohol in both males and females.

The rates of gambling reported in ALSPAC were lower than the British average during lockdown, probably because the sample was 71% female. During the same period as the ALSPAC COVID-19 questionnaires, YouGov were conducting online surveys during lockdown of 2000 British adults’ behaviour, including gambling (YouGov tracker, 2020). The seven waves of the YouGov survey conducted from 16th April to 18th June showed that past-four-week gambling participation by adults of all ages remained relatively stable, within a range of 28–32%. However, young adults aged 18–34 were significantly more likely than average to report increases in time or money spent on at least one gambling activity. Amongst regular ‘engaged’ gamblers, YouGov also found an increase in trying new online activities, such as online bingo, betting on virtual races or sports, National Lottery online and online slots.

The increase in online gambling seen in ALSPAC was not surprising considering the circumstances of lockdown, and consistent with the steady increase previously seen in the same respondents from 17 to 24 years in the use of online methods of gambling and betting (Hollén et al., 2020). As the increase in home working may well continue after the pandemic, it also possible that gambling online may continue to increase as a recreational activity for young adults spending more time at home. Gaming and gambling online are showing increasing convergence all over the world (Kim & King, 2020). The COVID-19 pandemic is likely to exacerbate existing inequalities that are already expressed in the distribution of gambling harms: a public health approach to the reduction of gambling related harms is called for (Griffiths et al 2020).

Data from the biggest gambling operators in the UK, covering 80% of the online gambling market (Gambling Commission, 2020), show that gross gambling yield increased during lockdown for online betting and online poker, but the biggest change was in betting online on e-sports, the income from which increased 3000% during the lockdown period between March-June 2020. Betting on e-sports was not separated in the ALSPAC COVID-19 questionnaire, but undoubtably increased as the usual outlets for sports betting (e.g. soccer) were suspended. A review of player data from a large European operator showed a decrease in the amount of money wagered by sports bettors during the COVID-19 pandemic (compared with before it) and that sports bettors did not switch to playing more online casino games. (Auer et al., 2020). In Sweden, those reporting sports betting during a period with decreased sports betting opportunities were shown to have markedly higher gambling problems (Håkansson, 2020). An online survey of regular sports betters in the UK during COVID lockdown (Wardle et al., 2021) has demonstrated that, while betting generally decreased, problem gambling was associated with starting new gambling activities during lockdown for men and that women who were shielding for health reasons were especially vulnerable to gambling harms.

The association between regular gambling and heavy regular alcohol use seen during lockdown has previously been observed in ALSPAC at 24 years (Hollén et al., 2020), and has been widely reported in the gambling literature (e.g. Scholes-Balog et al., 2016). The ALSPAC sample size was too small to look at alcohol intake change between age 24 years and during lockdown. The lack of association with mental health and well-being was surprising given the alcohol link, but previous studies have suggested that gambling and mood have a complex bi-directional relationship in young people (Chinneck et al., 2016; Dussault et al 2011).

The frequency of gambling during COVID-19 lockdown did not show any clear relationship with employment status: this may reflect the lockdown sample being well educated, as at the 24 year assessment, regular gambling in ALSPAC was associated with not being in education or employment (Hollén et al., 2020). The Health Survey for England in 2018 (NHS Digital, 2019) reported for adults over 16 years the following frequencies of any gambling by economic activity: employed 62%, retired 50%, in full time education 26%, unemployed 41%, other inactive 39%.

The gamblers in the ALSPAC study did report more financial difficulties pre- lockdown than non-gamblers, and some young people may have been gambling during lockdown in response to, or to escape from, financial hardship. However, the questionnaire data were not detailed enough to conclude any relationship with gambling spend.

The strengths of this study were the use of a well characterised cohort of young adults, some of whom had answered the same gambling questions 3 years previously, with contemporaneous self- report of mental health and well-being. The study also had some important limitations: although the sample size was large it was a self-selected, well-educated sub-group of the wider ALSPAC cohort; the majority of respondents were female, whereas males were more likely to be regular gamblers. Consequently, the associations shown were likely to underestimate the true extent of gambling activity during lockdown.

## Conclusions

Gambling online saw the biggest increase during lockdown, irrespective of gambling frequency. In regular gamblers, the proportion playing national lottery and doing private betting also increased but most other gambling activities remained stable or, for occasional gamblers, decreased in frequency. Previous gambling at aged 24, a history of financial difficulties pre-COVID, and heavy regular use of alcohol, were associated with regular gambling during lockdown.

## Appendix

See Table

**Table 6:** Gambling activities included in the COVID questionnaire

Activity	Include	Exclude
1. Lottery games	Thunderball and Euromillions	Scratchcards
2. Scratchcards	National Lottery scratchcard games played online	Newspaper or magazine scratchcards
3. Any other lottery tickets	Charity lotteries for hospices, sports or social clubs, e.g. "Monday Lottery"	Irish Lottery or any other international lotteries or buying raffle tickets
4. Football pools		Betting on football matches with a bookmaker
5. Bingo cards or tickets	Playing boards at a Bingo Hall	Newspaper bingo tickets, or bingo played on-line
6. Fruit (slot) machines		Quiz machines
7. Virtual gaming machines	Betting on virtual roulette, keno, bingo etc. in a bookmaker’s	Quiz machines
8. Table games	Roulette, dice or cards in a casino	Poker or casino games played online
9. Online gambling	Playing poker, bingo, slot machine style games, or casino games for money online through a computer, mobile phone or interactive TV	Bets made with online bookmakers or betting exchanges
10. Online betting with a bookmaker	Betting online through a computer, mobile phone or interactive TV on any event or sport	Bets made with a betting exchange or spread betting
11. Betting exchange	Peer to peer betting	
12. Betting on horse races	Betting on horse races with a bookmaker, by phone, or at the track. Also includes tote betting and betting on virtual horse races shown in a bookmaker’s	Bets made with on-line bookmakers or betting exchanges
13. Betting on dog races	Betting on dog races in a bookmakers’, by phone, or at the track. Also includes tote betting and betting on virtual dog races shown in a bookmaker’s	Bets made with online bookmakers or betting exchanges
14. Betting on other event or sport	Betting on any other event than horse or dog races or sport at the bookmakers, by phone or at the venue. Also includes Irish Lottery, 49 s, e-sports	Bets made with online bookmakers or betting exchanges or spread betting
15. Spread-betting		
16. Private betting	Playing cards or games for money with friends, family or colleagues	
17. Any other gambling form		

6.
